# Impact of the microbial derived short chain fatty acid propionate on host susceptibility to bacterial and fungal infections *in vivo*

**DOI:** 10.1038/srep37944

**Published:** 2016-11-29

**Authors:** Eleonora Ciarlo, Tytti Heinonen, Jacobus Herderschee, Craig Fenwick, Matteo Mombelli, Didier Le Roy, Thierry Roger

**Affiliations:** 1 Infectious Diseases Service and Department of Medicine, Centre Hospitalier Universitaire Vaudois and University of Lausanne, Epalinges, 1066, Switzerland; 2 Division of Immunology and Allergy, Department of Medicine, Centre Hospitalier Universitaire Vaudois and University of Lausanne, Epalinges, 1066, Switzerland

## Abstract

Short chain fatty acids (SCFAs) produced by intestinal microbes mediate anti-inflammatory effects, but whether they impact on antimicrobial host defenses remains largely unknown. This is of particular concern in light of the attractiveness of developing SCFA-mediated therapies and considering that SCFAs work as inhibitors of histone deacetylases which are known to interfere with host defenses. Here we show that propionate, one of the main SCFAs, dampens the response of innate immune cells to microbial stimulation, inhibiting cytokine and NO production by mouse or human monocytes/macrophages, splenocytes, whole blood and, less efficiently, dendritic cells. In proof of concept studies, propionate neither improved nor worsened morbidity and mortality parameters in models of endotoxemia and infections induced by gram-negative bacteria (*Escherichia coli, Klebsiella pneumoniae*), gram-positive bacteria (*Staphylococcus aureus, Streptococcus pneumoniae*) and *Candida albicans*. Moreover, propionate did not impair the efficacy of passive immunization and natural immunization. Therefore, propionate has no significant impact on host susceptibility to infections and the establishment of protective anti-bacterial responses. These data support the safety of propionate-based therapies, either via direct supplementation or via the diet/microbiota, to treat non-infectious inflammation-related disorders, without increasing the risk of infection.

Host defenses against infection rely on innate immune cells that sense microbial derived products through pattern recognition receptors (PRRs) such as toll-like receptors (TLRs), c-type lectins, NOD-like receptors, RIG-I-like receptors and cytosolic DNA sensors. The interaction of microbial ligands with PRRs activates immune cells to produce immunomodulatory molecules like cytokines and co-stimulatory molecules^1^,^2^,^3^. Pro-inflammatory cytokines play an essential role in coordinating the development of the innate and adaptive immune responses aimed at the eradication or containment of invading pathogens. Yet, inflammation has to be timely and tightly regulated since it may become life-threatening both by default and by excess^3^,^4^,^5^,^6^.

Short chain fatty acids (SCFAs) are end products of the fermentation of resistant starches and dietary fibers by intestinal bacteria, with the most abundant metabolites produced being acetate, propionate and butyrate^7^. SCFAs reach elevated concentrations in the gut lumen (50–100 mM) and are absorbed into the portal circulation, acting as a source of SCFAs in the bloodstream (0.1–1 mM)^8^,^9^,^10^,^11^. SCFAs, primarily butyrate, not only serve as a source of energy, but also stimulate neural and hormonal signals regulating energy homeostasis^12^. Beside their trophic effects, SCFAs possess antioxidative, anticarcinogenic and anti-inflammatory properties and play an essential role in maintaining gastrointestinal and immune homeostasis^7^,^10^,^11^,^13^.

Both extracellular and intracellular SCFAs exert immunosuppressive effects. Extracellular SCFAs act through metabolite sensing G-protein coupled receptors (GPCRs) such as GPR41, GPR43 and GPR109A^7^,^14^. Although conflicting results have been reported^13^,^15^, GPCRs were recently proposed to mediate the anti-inflammatory effects of SCFAs and protect form colitis, rheumatoid arthritis and airway hyper-responsiveness^16^,^17^,^18^,^19^. GPCRs propagate anti-inflammatory effects at least through a β2-arrestin-dependent stabilization of IκBα and inhibition of NF-κB-dependent transcription, and by promoting the generation of T regulatory cells (Tregs)^7^,^10^.

Upon diffusion into cells, intracellular SCFAs inhibit zinc-dependent histone deacetylases (i.e. HDAC1-11)^20^. HDACs are major epigenetic erasers catalyzing the deacetylation of histones, leading to chromatin compaction and transcriptional repression^21^. HDACs also target signaling molecules and transcription factors. Inhibitors of HDAC (HDACi) directly or indirectly impair NF-κB and Foxp3 activity, mediating anticancer, anti-neurodegenerative and anti-inflammatory activities, in part by inducing Treg generation^13^,^21^,^22^,^23^. Numerous HDACi are tested in clinical trials and several have reached the clinic. Besides valproate that is used since decades as a mood stabilizer and anti-epileptic, vorinostat, romidepsin and belinostat are used for the treatment of cutaneous and/or peripheral T-cell lymphoma, and panobinostat is used to treat patients with multiple myeloma who experienced two prior therapies^23^. HDACi are also viewed as promising latency-reversing agents to purge the HIV reservoir^24^. In agreement with their powerful anti-inflammatory properties, several HDACi interfere with the development of innate immune responses, protect against lethal sepsis, and increase susceptibility to infection^25^,^26^,^27^,^28^,^29^,^30^,^31^,^32^.

The development of SCFA-mediated therapies, either through direct supplementation with SCFAs or diet-induced modifications of the microbiota and production of endogenous SCFAs is an active area of research^16^,^17^,^18^,^19^,^33^. Considering that SCFAs carry anti-inflammatory activity and that HDACi were shown to increase susceptibility to infections in preclinical models and in patients enrolled in oncologic clinical studies^29^,^34^,^35^,^36^,^37^,^38^, an important question is whether SCFA-mediated therapies are safe. Here we focused on propionate as a representative SCFA reported to modulate adaptive immune responses *in vivo*^39^,^40^,^41^,^42^,^43^. We analyzed the response of macrophages, dendritic cells (DCs), splenocytes and whole blood to microbial compounds. Additionally, we performed proof of concept studies using a large panel of preclinical mouse models of endotoxemia, gram-positive and gram-negative bacterial and fungal infection of diverse severity. The results show that propionate to some extent inhibits innate immune responses *in vitro,* but does not alter susceptibility to infection *in vivo* nor inhibit passive or natural immunization. These data support the safety of therapies using propionate for treating non-infectious inflammation-related disorders.

## Results

### Impact of propionate on the response of immune cells to microbial stimulation

**Figure 1:**
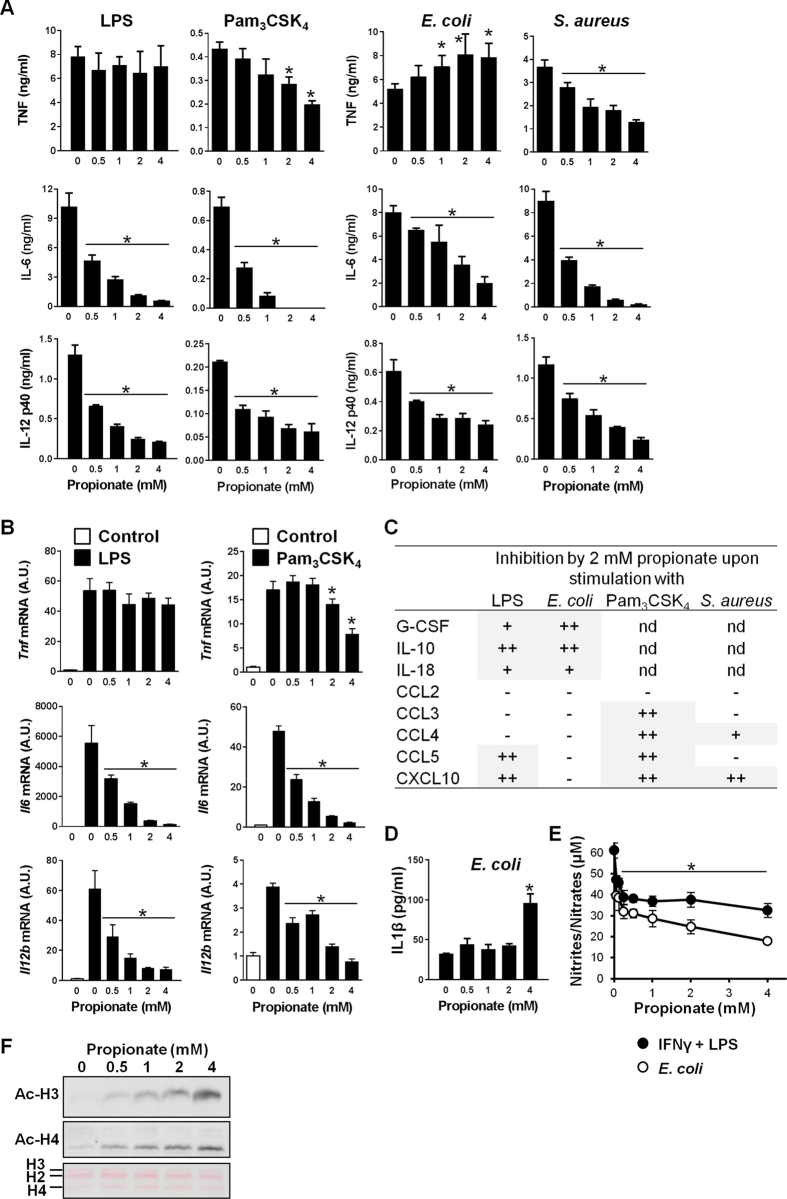
Impact of propionate on the response of macrophages to microbial stimulation. BMDMs were pre-incubated for 1 h with increasing concentrations (0, 0.06, 0.12, 0.25, 0.5, 1, 2 and 4 mM) of propionate before exposure for 4, 8 or 24 h to LPS (10 ng/ml), Pam_3_CSK_4_ (10 ng/ml), *E. coli* (10^6^ CFU/ml), *S. aureus* (10^7^ CFU/ml) or a combination of IFNγ (100 U/ml) plus LPS (10 ng/ml). (**A**,**B**) TNF, IL-6 and IL-12p40 concentrations in cell culture supernatants and *Tnf, Il6, Il12b* mRNA levels were quantified by ELISA (**A**, t = 8 h) and real time-PCR (**B**, t = 4 h). No cytokine was detected in the supernatants of unstimulated cells (*P* < 0.001 vs stimulus alone). *Tnf, Il6* and *Il12b* mRNA levels were normalized to *Hprt* mRNA levels. Data are means ± SD of triplicate samples from one experiment performed with 4 mice and representative of 2 experiments. *P < 0.05 vs stimulus without propionate. A.U.: arbitrary units. (**C**) The production of G-CSF, IL-10, IL-18, CCL2, CCL3, CCL4, CCL5 and CXCL10 was assessed by the Luminex technology (t = 8 h). Data summarize the impact of 2 mM propionate on mediators produced in response to LPS, *E. coli*, Pam_3_CSK_4_ and *S. aureus*: −, no inhibition; +, 1.5-2-fold inhibition; ++, >2-fold inhibition. Quantification is from one experiment performed with 4 mice. (**D**) IL-1β in cell culture supernatants. Data are means ± SD of triplicate samples from one experiment performed with 2 mice. *P < 0.05 vs no propionate. (**E**) Nitrites/nitrates were quantified using the Griess reagent (t = 24 h). Data are means ± SD of quadruplicate samples from one experiment performed with 4 mice. *P < 0.05 when comparing propionate at all concentrations vs no propionate. (**F**) Western blot analysis of acetylated histone 3 (Ac-H3) and Ac-H4 in BMDMs treated for 18 h with propionate. Ponceau staining of the membrane shows equal loading of total histones. Full-length blots are presented in Supplementary Figure S1.

To address the effects of propionate on the response of immune cells to microbial stimulation, bone marrow-derived macrophages (BMDMs) were exposed for 8 h to LPS (a TLR4 agonist), Pam_3_CSK_4_ (a lipopeptide triggering cells through TLR1/TLR2) and *Escherichia coli (E. coli*) and *Staphylococcus aureus (S. aureus*), used as representative gram-negative and gram-positive bacteria. The levels of TNF, IL-6 and IL-12p40 produced by BMDMs were quantified by ELISA (Fig. 1A). Propionate (0.5–4 mM) dose-dependently inhibited TNF production induced by Pam_3_CSK_4_ and *S. aureus*, and IL-6 and IL-12p40 production induced by LPS, Pam_3_CSK_4_, *E. coli* and *S. aureus*. Similar to other HDACi^29^,^44^,^45^, propionate did not inhibit TNF production induced by LPS and *E. coli*, and slightly amplified TNF response to *E. coli*. Accordingly, propionate powerfully inhibited LPS and Pam_3_CSK_4_-induced *Il6* and *Il12b* mRNA, to a lesser extent Pam_3_CSK_4_-induced *Tnf* mRNA, but not LPS-induced *Tnf* mRNA expression (Fig. 1B).

The anti-inflammatory activity of propionate was compared to that of butyrate and valproate by defining the IC_50_ of each of the SCFAs for LPS-induced IL-6 and IL-12p40 production. Similar IC_50s_ were obtained for IL-6 and IL-12p40: 0.01–0.05 mM for butyrate, 0.2–0.4 mM for valproate and 0.2–0.3 mM for propionate. Thus, propionate is as potent as valproate at inhibiting IL-6 and IL-12p40 but 8–20 fold less efficient than butyrate. The concentrations of G-CSF, IL-10, IL-18, CCL2/MCP-1, CCL3/MIP-1α, CCL4/MIP-1β, CCL5/RANTES and CXCL10/IP10 released by BMDMs exposed to LPS, *E. coli*, Pam_3_CSK_4_ and *S. aureus* were measured by Luminex (Fig. 1C). Whereas LPS and *E. coli* induced the secretion of all mediators, Pam_3_CSK_4_ and *S. aureus* did not induce the production of G-CSF, IL-10 and IL-18. Propionate inhibited G-CSF, IL-10 and IL-18 induced by LPS and *E. coli*, and CCL5 and CXCL10 induced by LPS. Propionate also inhibited CCL3, CCL4, CCL5 and CXCL10 induced by Pam_3_CSK_4_ and CCL4 and CXCL10 induced by *S. aureus*. Overall, propionate impaired more powerfully cytokine/chemokine secretion induced by Pam_3_CSK_4_ than LPS, like structurally unrelated HDACi^29^,^44^,^45^, and more efficiently cytokine production induced by pure microbial ligands than whole bacteria triggering similar PRRs (*i.e.* LPS vs *E. coli*, and Pam_3_CSK_4_ vs *S. aureus*). Notably, and in line with recent reports^46^,^47^, propionate at 4 mM increased *E. coli*-induced IL-1β secretion by BMDMs (Fig. 1D). Thus propionate impacts on inflammation in a cytokine dependent manner. Propionate also inhibited the production of nitric oxide (NO) induced by *E. coli* or IFNγ/LPS in BMDMs (50% inhibition using 0.6 mM and 4 mM propionate, respectively (Fig. 1E)).

To answer the question whether propionate acted through HDAC inhibition or via GPCRs, we first quantified mRNA levels of Hdac1-11 and free fatty acid receptor 2 (Ffar2) and Ffar3 encoding for GPR43 and GPR41. Ffar2 and Ffar3 mRNAs were not detected in BMDMs, in line with a previous report^41^. Incubation of BMDMs with propionate (0–4 mM for 4 or 18 hours) slightly modulated Hdac1-11 expression (range: 1.2–2.5 fold increase or decrease). Yet, propionate strongly increased histone 3 (H3) and H4 acetylation in a dose-dependent manner (Fig. 1F), indicating that propionate inhibits histone deacetylase activity in BMDMs.

**Figure 2:**
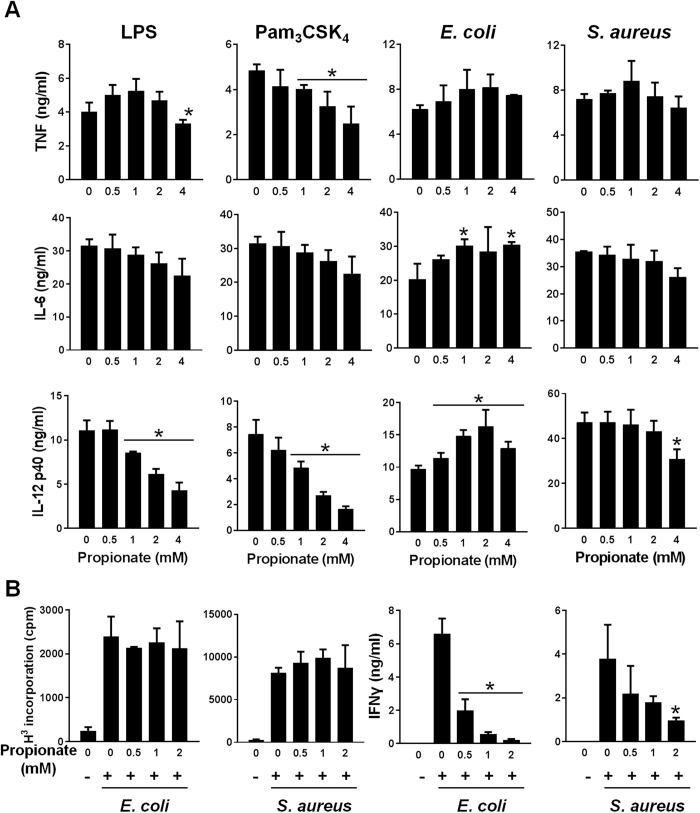
Impact of propionate on the response of dendritic cells and splenocytes. (**A**) BMDCs were pre-incubated for 1 h with increasing concentrations (0, 0.5, 1, 2 and 4 mM) of propionate before exposure for 8 h to LPS (10 ng/ml), Pam_3_CSK_4_ (10 ng/ml), *E. coli* (10^6^ CFU/ml) and *S. aureus* (10^7^ CFU/ml). TNF, IL-6 and IL-12p40 concentrations in cell culture supernatants were quantified by ELISA. Data are means ± SD of triplicate samples from one experiment performed with 4 mice and representative of 2 experiments. No cytokine was detected in the supernatants of unstimulated cells (*P* < 0.001 vs stimulus alone). (**B**) Mouse splenocytes were incubated for 48 h with or without propionate and *E. coli* or *S. aureus* (10^6^ CFU/ml). Proliferation was measured by ^3^H-thymidine incorporation. IFNγ concentrations in cell culture supernatants were quantified by ELISA. Data are means ± SD of triplicate samples from one experiment performed with 4 mice. *P < 0.05 vs stimulus without propionate.

Bone marrow-derived dendritic cells (BMDCs) were less sensitive than BMDMs to the anti-inflammatory effects of propionate. In BMDCs, propionate only significantly inhibited Pam_3_CSK_4_-induced TNF and IL-12p40 production in response to LPS, Pam_3_CSK_4_ or *S. aureus* (Fig. 2A). Of note, propionate slightly increased *E. coli*-induced IL-6 and IL-12p40 production by BMDCs. The viability of BMDMs and BMDCs incubated for 18 h with up to 8 mM propionate was greater than 98%, suggesting that propionate’s effects were not related to cytotoxicity. Along with a good tolerability of immune cells to propionate, propionate barely affected the proliferation of splenocytes exposed to *E. coli* and *S. aureus* whereas it efficiently inhibited IFNγ production (Fig. 2B).

**Figure 3:**
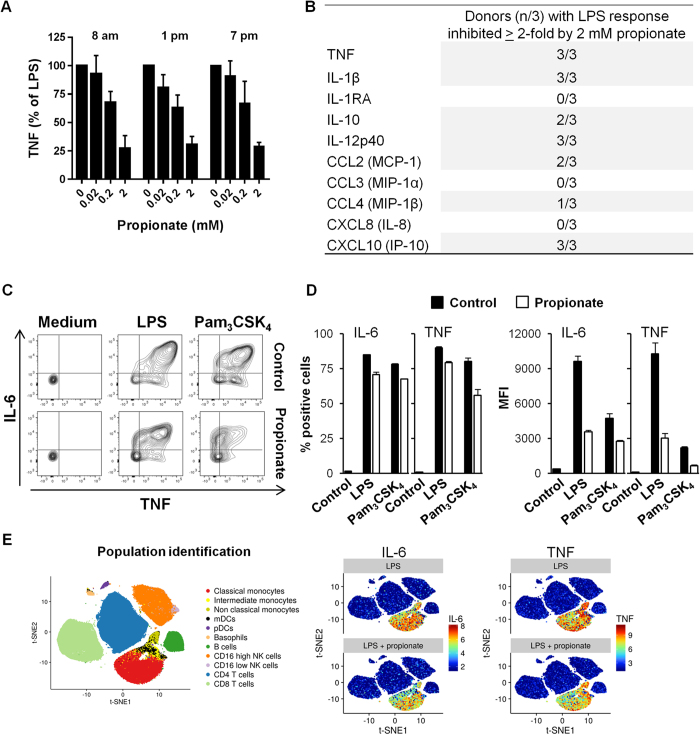
Impact of propionate on the response of human whole blood and monocytes. Whole blood from 3 healthy subjects was incubated for 18 h with propionate and LPS (100 ng/ml). (**A**) TNF released by whole blood collected at 8 am, 1 pm and 7 pm was quantified by ELISA. Data are expressed as the percentage of maximal (LPS without propionate) TNF release. No TNF was detected in the absence of LPS stimulation (not shown). Data are means ± SD from 3 healthy subjects. *P* < 0.005 when comparing 0.2 and 2 mM propionate with 0 mM propionate. (**B**) TNF, IL-1β, IL-1RA, IL-10, IL-12p40, CCL2, CCL3, CCL4, CXCL8 and CXCL10 were quantified by Luminex. Results summarize the number of donors in whom propionate inhibited significantly (P < 0.05) and by at least 2-fold cytokine release. (**C**,**D**) PBMCs were incubated for 1 h with 2 mM propionate and stimulated for 4 h with LPS (100 ng/ml) and Pam_3_CSK_4_ (1 μg/ml). TNF and IL-6 expression in CD14^+^ monocytes was analyzed by flow cytometry to calculate the percentage of positive cells (**C**) and mean fluorescence intensity (MFI) (**D**). Data are means ± SD from one experiment performed with 2 donors. (**E**) Whole blood incubated for 4 h with 2 mM propionate and 100 ng/ml LPS was fixed with Smart Tube stabilizer, and processed by CyTOF as described in *Materials and Methods*. Left: t-SNE scatter plot of non-granulocyte events. Right: t-SNE plot with arcsinh transformed signal intensity of IL-6 and TNF. Data are representative of results obtained with 3 donors.

The impact of propionate was tested on human cells. Propionate dose-dependently inhibited TNF production by whole blood exposed to LPS (Fig. 3A), albeit less efficiently than butyrate (TNF: 61 ± 6% vs 96 ± 4% and IL-6: 41 ± 7% vs 70 ± 10% inhibition using propionate vs butyrate at 2 mM, n = 3 donors collected at 8 am; *P* < 0.05). The extent of TNF inhibition by propionate was similar using blood collected at various times of the day (8 am, 1 pm and 7 pm), excluding a circadian rhythm-dependent effect. A Luminex quantification of 10 mediators produced by whole blood exposed to LPS extended to IL-1β, IL-10, IL-12p40, CCL2 and CXCL10 the spectrum of cytokines and chemokines whose expression was significantly inhibited (≥2-fold) by 2 mM propionate in at least 2 out of the 3 donors tested (Fig. 3B). In parallel experiments, butyrate inhibited more powerfully than propionate the secretion of IL-10 (in 3/3 vs 2/3 donors), CCL2 (3/3 vs 2/3) and CCL4 (2/3 vs 1/3). Butyrate also impaired the release of IL-1RA (3/3 donors) and CXCL8 (1/3). In a confirmation approach, flow cytometry analyses of intracellular cytokine expression in purified human CD14^+^ monocytes exposed to LPS and Pam_3_CSK_4_ revealed that propionate reduced the percentage (11–24% reduction) and the mean fluorescence intensity (1.7–3.4 fold reduction) of TNF and IL-6 positive cells (Fig. 3C and D). Additionally, mass cytometry (CyTOF^48^) analyses on human whole blood demonstrated that propionate inhibited IL-6 and TNF production by both classical and non-classical monocytes (Fig. 3E). Altogether, propionate inhibited, in a cell and stimulus-specific manner, the response of mouse and human immune cells *in vitro*. We next investigated the impact of propionate *in vivo*.

### Propionate does not protect from lethal endotoxemia and severe sepsis

Following common procedures used to study the impact of SCFAs *in vivo*^17^,^18^,^39^,^41^,^42^,^43^, mice were fed with propionate at 200 mM in the drinking water for 3 weeks, unless otherwise specified, before being used in preclinical models of toxic shock and infection. Attesting of the effectiveness of the treatment, 3 weeks of propionate regimen increased the number of splenic Foxp3^+^ Tregs (116% when compared to control mice; n = 8–11 animals per group; *P* = 0.0007) and of acetylated H4 in stomach, blood and bone marrow (3.5, 3.6 and 1.8 fold increased versus control mice, n = 2).

**Figure 4:**
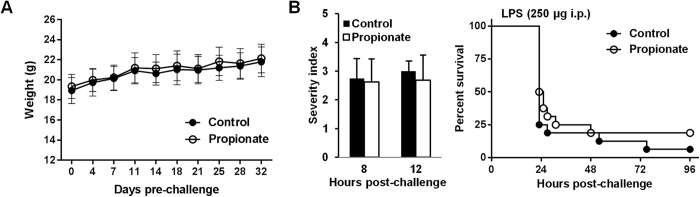
Propionate does not protect from lethal endotoxemia. BALB/c mice (n = 16 per group) were treated with or without 200 mM propionate in drinking water for 1 month. (**A**) Weight of animals under propionate treatment. (**B**) Severity scores (*P* > 0.1) and survival (*P* = 0.3) of mice challenged with LPS (250 μg i.p.).

Overwhelming inflammatory responses are deleterious for the host, and inhibition of the release of pro-inflammatory mediators confers protection in preclinical models of sepsis^3^,^4^,^5^. Moreover, several HDACi were shown to protect from toxic shock^49^. Therefore, we first tested propionate in a mouse model of acute endotoxemia. One month of propionate treatment had no impact on animal weight (Fig. 4A). In mice challenged with a lethal dose of LPS, severity scores and survival rates were similar whether or not animals were treated with propionate (*P* > 0.5 and *P* = 0.3; Fig. 4B).

**Figure 5:**
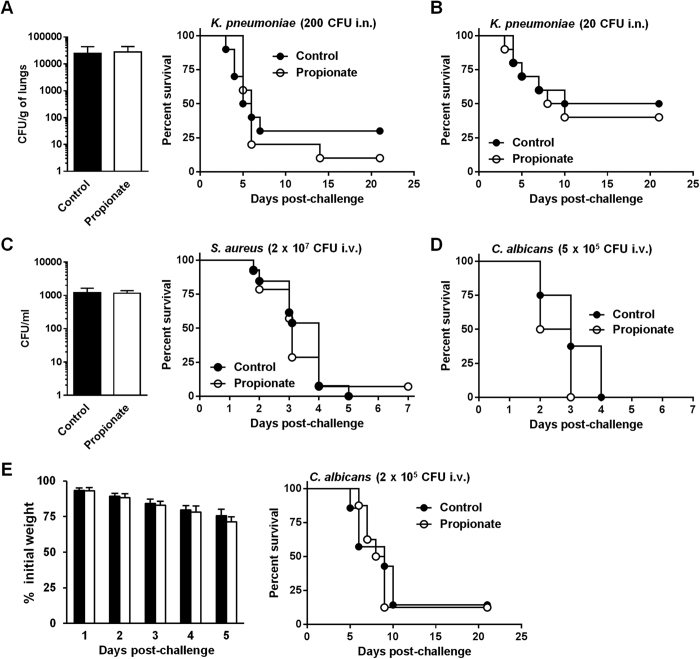
Propionate does not protect from lethal sepsis. BALB/c mice were treated with or without 200 mM propionate in drinking water for 3 weeks. (**A**,**B**) Bacterial counts in lungs 48 h post-infection and survival of mice (n = 10 per group) challenged i.n. with 200 CFU (**A**) or 20 CFU (**B**) of *K. pneumoniae. P* = 0.4, 0.8 and 0.7, respectively. (**C**) Bacterial counts in blood 24 h post-infection and survival of mice (n = 15 per group) challenged with *S. aureus* (2 × 10^7^ CFU i.v.). *P* = 0.9 and *P* = 0.6. (**D**,**E**) Survival and body weight of mice (n = 8 per group) challenged with *C. albicans* (5 × 10^5^ CFU i.v. in **D** and 2 × 10^5^ CFU i.v. in **E**). *P* = 0.1, *P* > 0.1 and *P* = 0.8, respectively.

The class of innate immune responses challenged by propionate was extended using models of severe sepsis induced by gram-negative (*Klebsiella pneumoniae, K. pneumoniae*), gram-positive (*S. aureus*) and fungal (*Candida albicans, C. albicans*) pathogens administrated either intranasally (i.n., *K. pneumoniae*) or intravenously (i.v., *S. aureus* and *C. albicans*). In mice challenged with 200 CFU *K. pneumoniae*, bacterial loads in lungs (*P* = 0.4) and mortality (70% vs 90% in control vs propionate groups; *P* = 0.8) were not affected by propionate (Fig. 5A). Mortality was also similar in control and propionate-treated mice infected with 20 CFU of *K. pneumoniae* (50% vs 60% in control vs propionate group; *P* = 0.7; Fig. 5B). In the severe model of systemic infection with *S. aureus*, bacterial counts in blood (*P* = 0.9) and mortality (100% vs 93% in control vs propionate groups; *P* = 0.6) were comparable with or without propionate treatment (Fig. 5C). In the acute model of candidiasis all mice died within 4 days, irrespective of the treatment applied (*P* = 0.1; Fig. 5D). The inoculum of *C. albicans* was then adjusted to produce a milder form of candidiasis during which mortality occurs 5 to 10 days after infection. Weight loss (*P* > 0.1) monitored during the first 5 days and survival (14.3% and 12.5%; *P* = 0.8) were comparable in untreated and propionate-treated mice (Fig. 5E).

**Figure 6:**
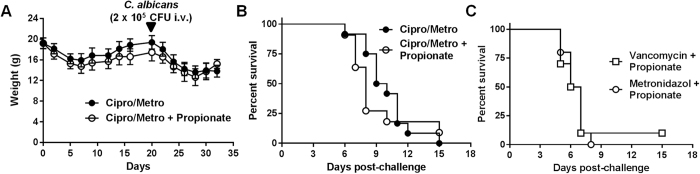
Propionate does not protect from candidiasis mice depleted of gut microbiota. BALB/c mice (n = 10 per group) were treated with ciprofloxacin (0.2 mg/ml) and metronidazole (1 mg/ml) or metronidazole or vancomycin (1 mg/ml) with or without 200 mM propionate in drinking water for 3 weeks and challenged with *C. albicans* (2 × 10^5^ CFU i.v.). (**A**) Body weight. (**B**,**C**) Survival of mice. *P* > 0.05.

Even though propionate was shown to impact on immune parameters of mice with a normal microbiota^17^,^18^,^39^,^43^,^45^,^50^, propionate produced by gut bacteria may attenuate the impact of propionate supplementation in models of infection. To address this issue, mice were treated with a combination of ciprofloxacin and metronidazole (CM) to deplete the gut flora and decrease endogenous SCFAs levels^39^,^51^. CM-treated mice lost 17% weight during the first week of treatment and recovered initial weight after 3 weeks. CM-treated mice were more sensitive to candidiasis (median survival time: 9.5 days for CM vs 11.5 days for controls mice run in parallel; n = 10 mice/group; *P* = 0.05). Co-treatment with CM plus propionate slightly increased weight loss and impaired weight rebound of uninfected mice (Fig. 6A). CM-treated mice died in between days 6 and 15 after *C. albicans* challenge, and propionate supplementation did not protect CM-treated mice from candidiasis (*P* = 0.4; Fig. 6B). To delineate the impact of gram-positive and gram-negative bacteria, mice were treated, together with propionate, with either metronidazole to target anaerobic gram-negative bacteria or vancomycin to target gram-positive bacteria. The two treatments resulted in identical survival profiles (Fig. 6C). Overall, propionate did not interfere with acute, lethal, bacterial and fungal infections.

### Propionate does not sensitize to mild infection

**Figure 7:**
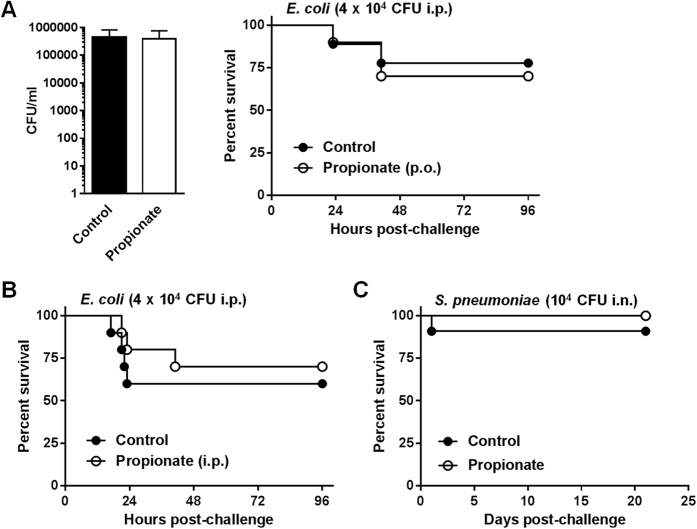
Propionate does not sensitize to mild infection by *E. coli* and *S. pneumoniae*. BALB/c mice were treated with or without 200 mM propionate in drinking water (**A**,**C**) or 1 g/kg propionate given i.p. every other day (**B**) for 3 weeks and challenged with *E. coli* (4 × 10^4^ CFU i.p.; n = 10 per group; (**A**,**B**) or *S. pneumoniae* (10^4^ CFU i.p.; n = 9–10; **C**). (**A**) Bacterial counts in blood 24 h post-infection and survival of mice. *P* = 0.9 and 0.7. (**B**,**C**) Survival of mice. *P* = 0.6 and 0.4.

Compromising innate immune responses may increase susceptibility to infection. To analyze the impact of propionate on mild infection, and to test another route of administration of propionate, propionate was given either *per os* or intraperitoneally (p.o.: 200 mM in water; i.p.: 1 g/kg i.p. every other day^43^) to mice subsequently challenged with *E. coli* titrated to cause a mild infection. Bacterial counts (*P* = 0.9) and survival rates (77% vs 70% and 60% vs 70% in control vs propionate groups upon p.o. and i.p. treatments; *P* = 0.7 and *P* = 0.6) were similar in all groups of treatment (Fig. 7A and B). Confirming that propionate does not sensitize mice to infection, 90% (9/10) of control mice and 100% (9/9) of propionate-treated mice infected i.n. with 10^4^ CFU *Streptococcus pneumoniae (S. pneumoniae*) survived infection (*P* = 0.4; Fig. 7C). Hence, propionate did not increase susceptibility to *E. coli* peritonitis and pneumococcal pneumonia.

### Propionate does not impair passive and natural immunization

**Figure 8:**
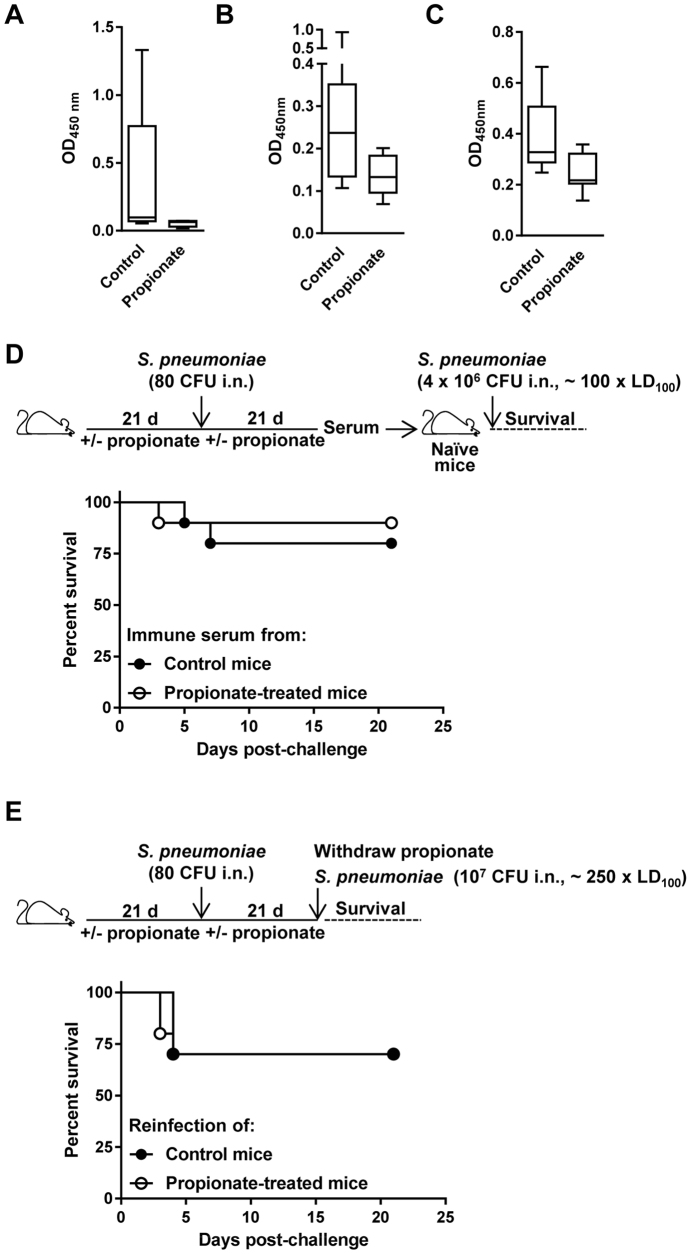
Propionate does not impair passive immunization and protection to secondary infection. Anti-*K. pneumoniae* (**A**), anti-*S. pneumoniae* (**B**) and anti-*C. albicans* (**C**) IgG titers in BALB/c mice surviving infection with 20 CFU *K. pneumoniae* (n = 4 control and 5 propionate-treated mice; Fig. 5B), 10^4^ CFU *S. pneumoniae* (n = 9 control and 10 propionate-treated mice; Fig. 7C) and 4 × 10^4^ CFU *C. albicans* (n = 9 control and 9 propionate-treated mice, serum was collected 3 weeks post-infection). Box and min-to-max whisker plots represent the OD_450 nm_ using plasma (diluted 1/200) collected on day 21 after infection. *P* = 0.1, 0.01 and 0.02, respectively. No signal was detected using plasma from uninfected mice. (**D,E**) BALB/c mice (n = 18–21 per group) were treated with or without 200 mM propionate in drinking water for 3 weeks, challenged i.n. with 80 CFU *S. pneumoniae*, and used for subsequent experimentation 3 weeks later. (**D**) Sera collected from 8 water and 11 propionate-treated mice were pooled and transferred (120 μl i.p.) into naive mice (n = 10 per group) infected 24 h later with 4 × 10^6^ CFU *S. pneumoniae* (~100 x LD_100_). Survival was monitored for 21 days. *P* = 0.6. (**E**) Propionate treatment was withdrawn. Mice (n = 10 per group) were infected with 10^7^ CFU *S. pneumoniae* (~250 x LD_100_). Survival was monitored for 21 days. *P* = 0.8.

We measured anti-*K. pneumoniae* and anti-*S. pneumoniae* IgG titers in mice surviving infection with 20 CFU *K. pneumoniae* (4 controls and 5 propionate-treated mice; Fig. 5B) and 10^4^ CFU *S. pneumoniae* (9 controls and 9 propionate-treated mice; Fig. 7C). Anti-bacterial IgG titers were reduced in propionate-treated mice (*P* = 0.1 and 0.01 for anti-*K. pneumoniae* and *S. pneumoniae* IgG titers, respectively; Fig. 8A and B). To confirm this observation, we measured IgG titers in mice infected 3 weeks earlier with a non-lethal inoculum of *C. albicans* (2 × 10^4^ CFU i.v.). Anti-*C. albicans* IgG titers were reduced in propionate fed mice (*P* = 0.02; Fig. 8C). In addition, splenic Foxp3^+^ Tregs were increased in propionate treated and *C. albicans* infected mice (113% when compared to control mice; n = 10 mice per group; *P* = 0.006). Therefore, although propionate did not interfere with morbidity and mortality in the models of infection presented above, it impacted to some extent on anti-microbial host responses. Two approaches were used to assess the relevance of this observation. Mice treated with or without propionate for 3 weeks were challenged with a non-lethal inoculum of *S. pneumoniae* (80 CFU). In a first setting, 3 weeks after infection, sera were collected and transferred into naive, non-treated, mice that were infected 24 h later with *S. pneumoniae* used at around 100 x LD_100_ (Fig. 8D). In a second setting, 3 weeks after infection, propionate treatment was withdrawn and mice were re-challenged with *S. pneumoniae* used at around 250 x LD_100_ (Fig. 8E). Overall, propionate treatment during the primary infection had no impact on outcome, and both transfer of immune serum and pre-exposure to a low *S. pneumoniae* inoculum protected mice from a lethal *S. pneumoniae* inoculum.

## Discussion

The gut microbiota and its metabolites exert strong influences on human health. Among bacterial metabolites, SCFAs have attracted much attention because of their beneficial influence on the development of inflammation-related pathologies in combination with the fact that their production can be influenced by the diet^7^,^10^,^52^,^53^. Here we show that propionate has powerful yet selective anti-inflammatory activity *in vitro*, and that it does not have a major impact on host susceptibility to infection *in vivo*. This observation is particularly relevant in light of the development of diet or microbiota targeting strategies to treat immune related diseases.

Propionate impaired cytokine production by innate immune cells, albeit differently according to the cell type, the microbial trigger and the cytokine analyzed. Similar disparities have been observed with other SCFAs^29^,^40^,^45^,^46^,^54^. BMDCs were more resistant to propionate than BMDMs, human monocytes and whole blood. In human monocyte-derived DCs (moDCs) and BMDCs, propionate modestly affected IL-6 but efficiently limited IL-12p40 production induced by LPS (ref. 40 and Fig. 2). Furthermore, propionate did not inhibit MHC-II and CD86 expression but impaired CD83 expression by moDCs^40^. Disparate cell responses to propionate may reflect, at least in part, differential expression of GPCRs. In depth analyses of the pattern and the expression levels of cell-surface GPCRs by immune and non-immune cells is still missing, and could give clues about the contradictory findings reported concerning the inflammatory phenotype of GPR41 and GPR43 knockout mice^15^. Moreover, SCFA specificity of GPCRs and how redundant behave GPCRs *in vitro* and *in vivo* are largely unresolved issues. For example, mice deficient in either GPR43 or GPR109A were susceptible to gut inflammation and developed exacerbated colitis, and mice deficient in either GPR41 or GPR43 were susceptible to allergic airway inflammation^17^,^18^,^19^,^43^,^55^. At least in the gut, expression of both GPR43 and GPR109A by non-hematopoietic cells contributed to the protective effects of high-fiber regimen against colitis^19^.

Besides acting through GPCRs, SCFAs act as inhibitors of class I and II HDACs (HDACi). HDACi impair innate and adaptive immune responses at multiple levels, including TLR and IFN signaling, cytokine production, bacterial phagocytosis and killing, leukocyte adhesion and migration, antigen presentation by DCs, cell proliferation and apoptosis, and Treg development and function^21^,^49^,^56^. In T cells, acetate, propionate and butyrate suppressed HDAC activity independently of GPR41 and GPR43^41^. Whether SCFAs mediate HDAC inhibition through GPCRs is debatable^13^, but it is worth mentioning that GPCR signaling modulates kinase, redox and acetylation pathways that, in turn, influence the cellular distribution and activity of histone acetyl transferases and HDACs^57^.

Like other structurally unrelated HDACi (trichostatin A and suberanilohydroxamic acid, *i.e.* vorinostat), acetate, propionate and butyrate increased acetylation of FOXP3 and potentiated the generation of peripheral Treg cells^39^,^42^,^58^. Moreover, SCFAs were recently reported to promote the generation of Th1 and Th17 cells during *Citrobacter rodentium* infection, suggesting a complex, context-dependent impact of SCFAs on immune responses^41^. Butyrate is a more potent HDACi than propionate, which is more potent than acetate. This ranking parallels the effectiveness of the anti-inflammatory activity of SCFAs^39^,^59^,^60^. Propionate failed to inhibit TNF but not IL-6 and IL-12p40 induced by LPS in BMDMs, which mirrored previous observations obtained with trichostatin A and suberanilohydroxamic acid^29^,^44^,^61^,^62^,^63^. Propionate strongly increased H3 and H4 acetylation in BMDMs which, like BMDCs, barely expressed *Ffar2* and *Ffar3*^41^. Thus, the effect of propionate on BMDMs is at least in part mediated by inhibition of HDACs. The fact that propionate slightly increased cytokine production under certain conditions is reminiscent of the paradoxical impact of HDACi on TNF secretion in human macrophages^46^. Further work will be required to unravel how propionate differentially affects cytokine expression induced by pure microbial ligands versus whole bacteria, for example by analyzing chromatin structure, modifications and activation of transcriptional regulators, and signaling pathways.

Acetate, propionate and butyrate are found at molar ratios of 60/20/20 in the intestinal tract and 90-55/35-5/10-4 in blood, depending on portal, hepatic and peripheral origins, where they altogether reach 50–150 mM and 0.1–1 mM, respectively^8^,^43^. The high plasma concentrations of propionate compared to butyrate may counterbalance its weaker anti-inflammatory activity. Further work will be required to analyze the effects of combinational treatments with SCFAs on innate immune cells. Propionate is produced primarily by Bacteroidetes via the succinate pathway and some Firmicutes through the lactate and succinate pathways, acetate by enteric bacteria and butyrate by Firmicutes^10^. SCFAs themselves modify the composition of the gut microbiota. Propionate stimulates the growth of *Bifidobacterium*^64^. Proportions of Bacteroidaceae and Bifidobacteriaceae increased in the gut of mice fed with a high-fiber diet, elevating acetate and propionate levels but decreasing butyrate concentrations in cecal content and blood^43^. Thus, changing microbiome composition affects SCFA levels both locally and systemically. Moreover, the gut microbiota protects from pneumococcal pneumonia^65^. In the perspective of targeting the diet or the microbiota for treating inflammatory conditions^7^,^10^,^52^,^53^, it was of prime interest to analyze the impact of propionate in preclinical models of infection.

Unlike powerful broad-spectrum HDACi^27^,^29^,^66^,^67^, propionate had no obvious impact on morbidity and mortality parameters in models of endotoxemia and infections. This contrasts with the effectiveness of SCFAs at ameliorating the clinical outcome in chronic inflammatory diseases like rheumatoid arthritis, colitis and airway allergy^17^,^18^,^19^,^33^,^39^,^43^,^45^,^50^. The propionate regimen itself was unlikely responsible of the failure to protect septic animals since it increased H4 acetylation in organs and identical or shorter treatments had an immune impact^39^,^43^. Moreover, as expected^39^,^43^,^50^, propionate increased the frequency of peripheral Tregs and reduced anti-microbial IgG responses, indicating that propionate influenced immune parameters during the course of infections. Multiple mechanisms may account for the reduced humoral response. Besides increasing the frequency of Tregs, SCFAs and HDACi have been shown to inhibit development and migration of DCs. Moreover, they decreased expression of costimulatory and MHC molecules, reduced production of T-cell polarizing factors and increased production of indoleamine 2,3-dioxygenase (a negative regulator of T-cell activation) by DCs. In mouse models, HDACi inhibited antigen presentation and allogeneic and syngeneic responses and decreased antibody generation^29^,^40^,^44^,^56^.

Albeit surprising at first glance, propionate did not increase the mortality of mice subjected to sub-lethal/mild infections. Indeed, one of the possible collateral damages of administrating immunomodulatory compounds is an increased risk of infections. A well-known example is anti-TNF therapies that are associated with reactivation of latent tuberculosis and viral infections as well as an increased risk of opportunistic infections^68^. Moreover, episodes of severe infection have been reported in patients treated with HDACi^34^,^35^,^36^,^37^,^38^. In the present study, we tested models of systemic and local infections using the most common etiologic agents of bacterial sepsis in humans (*E. coli, S. aureus* and *K. pneumoniae*) to investigate the safety of propionate supplementation for clinical purposes.

The production of propionate by intestinal bacteria has been proposed to represent a mechanism through which the host response to commensals is kept under control and avoid local inflammation and tissue damage. It is however now well established that SCFAs have much broader effects on human health. Using several preclinical mouse models, we report that administration of propionate neither protects from lethal sepsis nor increases susceptibility to mild infections. These results are encouraging in the perspective of developing propionate-based therapies, e.g. direct supplementation or via the diet/microbiota, without putting patients at risk of developing infections.

## Materials and Methods

### Ethics statement

Animal experimentations were approved by the Service de la Consommation et des Affaires vétérinaires (SCAV) du Canton de Vaud (Epalinges, Switzerland) under authorizations n° 876.7, 876.8, 877.7 and 877.8, and performed according to our institutional guidelines and ARRIVE guidelines (http://www.nc3rs.org.uk/arrive-guidelines). Experiments on human whole blood samples were carried out in accordance with guidelines and regulations of the Swiss Ethics Committees on research involving humans. The procedure, using anonymized human whole blood samples from healthy subjects without possibility to trace back subject identity, did not require prior ethics committee authorization. Written informed consent was obtained from blood donors at the Infectious Diseases Service, CHUV, Lausanne.

### Mice, cells and reagents

Female BALB/cByJ mice (8–10 week-old; Charles River Laboratories, Saint-Germain-sur-l’Arbresle, France) were housed under specific pathogen-free conditions. Bone marrow cells were cultured for 7 days in IMDM containing 50 μM 2-ME and 30% L929 supernatant as a source of M-CSF to generate bone marrow-derived macrophages (BMDMs), or GM-CSF to generate bone marrow-derived dendritic cells (BMDCs)^69^. Splenocytes were cultured in RPMI 1640 medium containing 2 mM glutamine and 50 μM 2-ME^70^. Culture media (Invitrogen, San Diego, CA) were supplemented with 10% (v/v) heat-inactivated FCS (Sigma-Aldrich St. Louis, MO), 100 IU/ml penicillin and 100 μg/ml streptomycin (Invitrogen). Propionate and butyrate were from Sigma-Aldrich, valproate from Desitin (Hamburg, Germany), *Salmonella minnesota* ultra pure lipopolysaccharide (LPS) from List Biologicals Laboratories (Campbell, CA) and Pam_3_CSK_4_ from EMC microcollections (Tübingen, Germany). *E. coli* O18:K1:H7 (*E. coli*), *K. pneumoniae* caroli (*K. pneumoniae*), *S. aureus* AW7 (*S. aureus*), *S. pneumoniae* 6303 (*S. pneumoniae*), and *C. albicans* were isolated from septic patients^71^,^72^. *E. coli, K. pneumoniae, S. aureus* and *S. pneumoniae* were grown in brain heart infusion broth, *C. albicans* in yeast extract-peptone-dextrose (BD Biosciences, Erembodegem, Belgium). For *in vitro* experiments, microorganisms were heat-inactivated for 2 h at 56 °C before usage.

### Cell viability assay

Cell viability was assessed using the 3-[4,5-dimethylthiazol-2-yl]-2,5-diphenyltetrazolium bromide (MTT) Cell Proliferation and Viability Assay and a Synergy H1 microplate reader (BioTek, Winooski, VT)^73^. On each 96-well cell culture plate, serial quantities of cells (0.3 × 10^4^–5 × 10^5^) were seeded to establish a standard curve.

### Whole blood assay

Heparinized whole blood (50 μl) obtained from healthy subjects was diluted 5-fold in RPMI 1640 medium and incubated with or without propionate and microbial products in 96-wells plates. Reaction mixtures were incubated for 24 h at 37 °C in the presence of 5% CO_2_. Cell-free supernatants were stored at −80 °C until cytokine measurement.

### Cytokine and NO measurements and flow cytometry analyses

Cell culture supernatants and plasma were used to quantify the concentrations of TNF, IL-1β, IL-6, IL-12p40 and IFN-γ by DuoSet ELISA kits (R&D Systems, Abingdon, UK), cytokines/chemokines using mouse (G-CSF, IL-1β, IL-10, IL-12p70, IL-18, CCL2/MCP1, CCL3/MIP-1α, CCL4/MIP-1β, CCL5/RANTES, CXCL10/IP10) and human (TNF, IL-1β, IL-1ra, IL-10, IL-12p40, CCL2, CCL3, CCL4, CXCL8/IL-8, CXCL10) Luminex assays (Affimetrix eBioscience, Vienna, Austria), and NO using the Griess reagent^71^,^74^. Intracellular cytokine staining was performed essentially as described^75^. Half a million peripheral blood mononuclear cells (PBMCs) were incubated for 1 h with propionate and then for 4 h with 1 μg/ml brefeldin A (BioLegend, San Diego, CA) with or without LPS (100 ng/ml) or Pam_3_CSK_4_ (1 μg/ml). PBMCs were stained with the LIVE/DEAD Fixable Aqua Dead Cell Stain Kit (Molecular Probes, ThermoFisher Scientific, Zug, Switzerland), washed with Cell Stain Medium (CSM: PBS, 0.5% BSA, 0.02% NaN_3_ and 2 mM EDTA), incubated with Human TruStain FcX™ (BioLegend) to block Fc receptors and stained with anti-CD3-PerCP/Cy5.5 (clone UCHT1) and anti-CD14-Pacific Blue (clone M5E2). Cells were washed with CSM, fixed using 2.4% (w/v) formaldehyde in PBS, washed with CSM-S (CSM containing 0.3% saponin, Sigma-Aldrich) and stained with anti-IL-6-allophycocyanin and anti-TNF-phycoerythrin/Dazzle™ 594 antibodies (clones MQ2-13A5 and MAB11). Single cell suspensions of splenocytes were stained with anti-CD4-APC/Cy7 (clone Gk1.5) and anti-CD25-APC (clone 3C7). Staining steps were performed at 20 °C for 20 min. All antibodies were from BioLegend. Foxp3 staining (anti-Foxp3-PE, clone FJK-16S,) was performed with the Foxp3/Transcription Factor Staining Buffer Set from eBioscience according to the manufacturer’s instructions. Unstained and single stained samples were used to calculate compensation for PBMCs while UltraComp eBeads (eBioscience) were used for splenocytes. Acquisition was performed on a LSR II flow cytometer (BD Biosciences). Foxp3 and CD25 gates were determined using FMO controls. Data was analyzed using FlowJo vX (FlowJo LCC, Ashland, OR).

### RNA analyses by real-time PCR

Total RNA was isolated, reverse transcribed and used for real-time PCR analyses using a QuantStudio™ 12 K Flex system (Life Technologies, Carlsbad, CA). Reactions consisted of 1.25 μl cDNA, 1.25 μl H_2_O, 0.62 μl primers and 3.12 μl Fast SYBR^®^ Green Master Mix (Life Technologies). Primer pairs for amplifying *Tnf, Il6, Il12b* and *Hprt (hypoxanthine guanine phosphoribosyl transferase*) cDNA were as published^69^. Samples were tested in triplicates. Gene specific expression was normalized to *Hprt* expression and expressed relative to that of untreated cells.

### Western blot analyses

Histones were extracted from cells or organs (disrupted using a gentleMACS^TM^ Octo Dissociator, Miltenyi Biotec; Bergisch Gladbach, Germany), run through SDS-PAGE and detected by Western blotting^29^ using antibodies (diluted 1:1000) directed against acetylated histone 3 (H3) and H4 (06-599, 06-860) (EMD Millipore, Billerica, MA). Blots were revealed with the enhanced chemiluminescence Western blotting system (GE Healthcare, Little Chalfont, Great Britain). Images were recorded using a Fusion Fx system (Viber Lourmat, Collégien, France).

### Proliferation assay

The proliferation of splenocytes (1.5 × 10^5^ cells) cultured for 48 h in 96-well plates was assessed by measuring ^3^H-thymidine incorporation over 18 h using a β-counter (Packard Instrument Inc, Meriden, CT).

### Mass cytometry (CyTOF) analysis

Blood was collected from healthy donors in heparin tubes and stimulated with LPS (100 ng/ml) with or without propionate (2 mM) and brefeldin A. After 4 h, EDTA (2 mM) was added and cells incubated for 15 min at room temperature (RT). Cells were fixed using a proteomic stabilizer (Smart Tube Inc., San Carlos, CA) and stored at −80 °C. Thawing and erythrocyte lysis was performed according to instructions from Smart Tube Inc. Fc receptors were blocked with Human TruStain FcX Receptor Blocker. Cells were stained using metal-conjugated antibodies according to the CyTOF manufacturer’s instructions (Fluidigm, South San Franciso, CA). Briefly, individual samples were stained with unique combinations of CD45 antibodies for 30 min at RT, followed by 3 washes with CSM. Samples were pooled and stained using a cocktail of antibodies for cell surface markers, washed with CSM and PBS, fixed with 2.4% formaldehyde, washed with CSM-S, and stained for intracellular targets. Antibodies directed against CD1c (L161), CD3 (UCHT1), CD4 (RPA-T4), CD7 (CD7-6B7), CD8 (SK1), CD11c (Bu15), CD14 (M5E2), CD16 (3G8), CD20 (2H7), CD45 (HI30), CD66b (G10F5), CD123 (6H6), HLA-DR (L243), IL-6 (MQ2-13A5) and TNF (Mab11) were from BioLegend, Slan (DD1) from Miltenyi Biotec and CD56 (R19-760) and CD141 (1A4) from BD. After intracellular staining, cells were resuspended in DNA-intercalation solution (PBS, 1 μM Ir-Intercalator, 1% formaldehyde, 0.3% saponin) and stored at 4 °C until analysis. For analysis, cells were washed 3 times with MilliQ water and resuspended at 0.5 × 10^6^ cells/ml in 0.1% EQ™ Four Element Calibration Beads solution (Fluidigm). Samples were acquired on an upgraded CyTOF 1 using a syringe pump at 45 μl/min. FCS files were concatenated and normalized using the cytobank concatenation tool and matlab normalizer respectively^76^. Data was processed and analyzed with cytobank and R using the OpenCyto and cytofkit packages^77^,^78^. Dimensionality reduction with t-SNE was performed on a merged dataset, consisting of a random selection of 10’000 non granulocyte events from each sample.

### *In vivo* models

Mice (n = 8–16/group) treated or not with propionate (200 mM in drinking water or 1 g/kg i.p. every other day) were challenged with LPS (250 μg i.p.), *E. coli* (4 × 10^4^ CFU i.p.), *K. pneumoniae* (20 or 200 CFU i.n.), *S. pneumoniae* (80, 10^4^, 4 × 10^6^ or 10^7^ CFU i.n.), *S. aureus* (2 × 10^7^ CFU i.v.) or *C. albicans* (4 × 10^4^, 2 × 10^5^ or 5 × 10^5^ conidia i.v.)^29^,^70^,^71^,^79^,^80^,^81^. Unless specified, propionate treatment was continued after microbial challenge. To analyze the impact of propionate on acquired immunity to bacterial infection, BALB/c mice with or without propionate treatment were infected with 80 CFU *S. pneumoniae*. Three weeks later, propionate treatment was stopped. Mice were either re-infected with 10^7^ CFU *S. pneumoniae* or sacrificed to collect sera. Sera from water or propionate-treated mice were pooled and transferred (120 μl i.p.) into naïve mice that were infected 24 h later with 4 × 10^6^ CFU *S. pneumoniae*. In selected experiments, mice were treated with ciprofloxacin (0.2 mg/ml; Fresenius, Brézins, France), metronidazole (1 mg/ml; Sintetica S.A., Couver, Switzerland) and vancomycin (TEVA, North Wales, PA) in drinking water to deplete the gut microbiota^17^,^18^,^39^,^43^,^82^. Body weight, severity scores and survival were registered at least once daily as described previously^71^.

### Detection of anti-bacterial and anti-*Candida* IgG by ELISA

Briefly, 96-well plates (Maxisorp, Affimetrix eBioscience) were coated with 5 × 10^6^ heat-killed *K. pneumoniae* or *S. pneumoniae* or 100 μg/ml *C. albicans* in bicarbonate/carbonate buffer (100 mM, pH 9.6), blocked with PBS containing 3% BSA (PBS-BSA) and incubated with mouse serum diluted 1/200 in PBS-BSA. IgGs were detected with peroxidase-goat anti-mouse IgG (H+L) and then 3,3′,5,5′-tetramethylbenzidine (TMB) Substrate Solution (ThermoFisher Scientific). Reactions were stopped using 0.16 M sulfuric acid and absorbance measured at 450 nm using a VersaMax ELISA microplate reader (Molecular devices, Sunnyvale, CA). All washing steps were performed using PBS containing 0.05% (v/v) Tween-20.

### Statistical analyses

Comparisons between the different groups were performed by analysis of variance followed by two-tailed unpaired Student’s t-test or Mann-Whitney test when appropriate. The Kaplan-Meier method was used for building survival curves and differences were analyzed by the log-rank sum test. All analyses were performed using PRISM (GraphPad Software). *P* values are two-sided, and values < 0.05 were considered to indicate statistical significance.

## Additional Information

**How to cite this article**: Ciarlo, E. *et al*. Impact of the microbial derived short chain fatty acid propionate on host susceptibility to bacterial and fungal infections *in vivo. Sci. Rep.* **6**, 37944; doi: 10.1038/srep37944 (2016).

**Publisher's note:** Springer Nature remains neutral with regard to jurisdictional claims in published maps and institutional affiliations.

## Supplementary Material

Supplementary Information
