# Supplementary figures and images for: Impact of the microbial derived short chain fatty acid propionate on host susceptibility to bacterial and fungal infections in vivo
Source: Sci Rep. 2016 Nov 29;6:37944. doi: 10.1038/srep37944 (PMC5126587; doi:10.1038/srep37944)

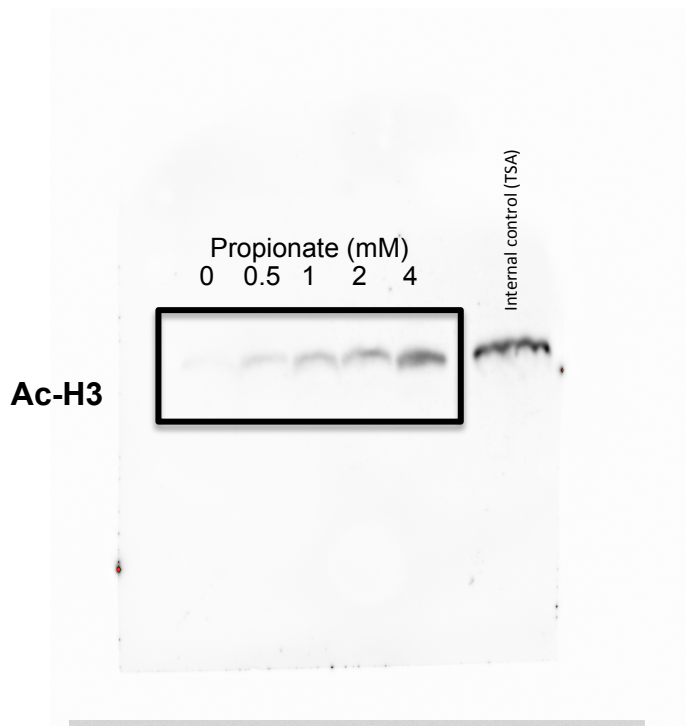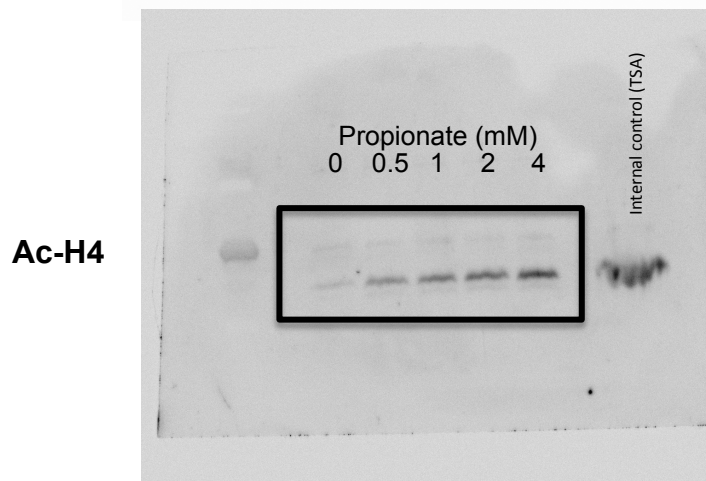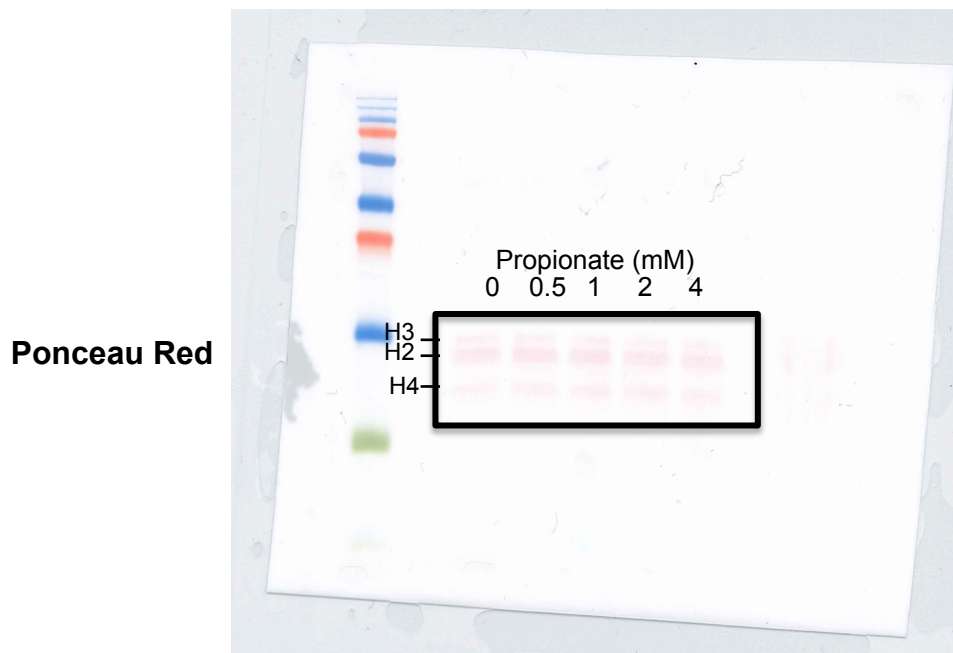

Supplement: Supplementary Information [file srep37944-s1.pdf]
